# Exposure to *Neospora* spp. and *Besnoitia* spp. in wildlife from Israel

**DOI:** 10.1016/j.ijppaw.2018.08.002

**Published:** 2018-08-17

**Authors:** Monica L. Mazuz, Gema Alvarez-García, Roni King, Igor Savisky, Varda Shkap, Luis M. Ortega-Mora, Daniel Gutiérrez-Expósito

**Affiliations:** a Parasitology Division, Kimron Veterinary Institute, PO Box 12, Bet Dagan, 50250, Israel; b SALUVET, Animal Health Department, Faculty of Veterinary Sciences, Complutense University of Madrid, Ciudad Universitaria s/n, 28040, Madrid, Spain; c Science Division, Israel Nature & Parks Authority, 3 Am Ve´Olamo St, Jerusalem, 9546303, Israel

**Keywords:** *Neospora caninum*, *Besnoitia besnoiti*, Wildlife, Serological survey, Western blot, Israel

## Abstract

Neosporosis and besnoitiosis, caused by cyst-forming protozoa *Neospora caninum* and *Besnoitia besnoiti*, respectively, are parasitic infestations of livestock in Israel. These parasites cause significant economic losses in cattle due to reproductive and productive disorders. Both parasites have been detected in several wild ruminant species throughout other regions of the world, while the existence of a sylvatic life cycle in Israel remains uncertain. Thus, a wide panel of 871 sera from two wild carnivores and nine wild ruminant species were tested. All sera were first analysed by MAT for an initial screening and positive samples were confirmed *a posteriori* by Western blot. Additionally, a complementary IFAT was used for the detection of antibodies against *N. caninum*. *Neospora* antibodies were present in six out of the 11 species investigated, whereas *Besnoitia* antibodies were undetected. Golden jackal, red fox, addax, Arabian oryx, Persian fallow deer, mouflon, mountain gazelle, Nubian ibex, scimitar horned oryx and water buffalo were seropositive against *N. caninum* infection by IFAT and/or MAT. Moreover, the presence of *Neospora* spp.-specific antibodies was confirmed by Western blot in golden jackal (6/189; 3.2%), red fox (1/75; 1.3%), Persian fallow deer (13/232; 5.6%), mouflon (1/15; 16.7%), Nubian ibex (22/55; 40%) and water buffalo (12/18; 66.7%). Addax (1/49) and water buffalo (1/18) were MAT-seropositive against *B. besnoiti* but were seronegative by Western blot. Hence, *Neospora* sylvatic cycle is present in Israel and may cross over to a domestic life cycle. In contrast, wildlife species investigated are unlikely to present a risk of transmitting *Besnoitia* to livestock in Israel.

## Highlights

• Two wild carnivore and nine ruminant species sera (*n* = 871) from Israel were tested.
• IFAT and MAT were used as screening tests and positive sera were confirmed by Western blot.
• Golden jackal, red fox, Persian fallow deer, mouflon, Nubian ibex and water buffalo were *Neospora* spp. positive.
• *Besnoitia* spp.-specific antibodies were not found.
• Two complementary diagnostic tests is a need to obtain more accurate results.

## Graphical abstract

**Figure:**
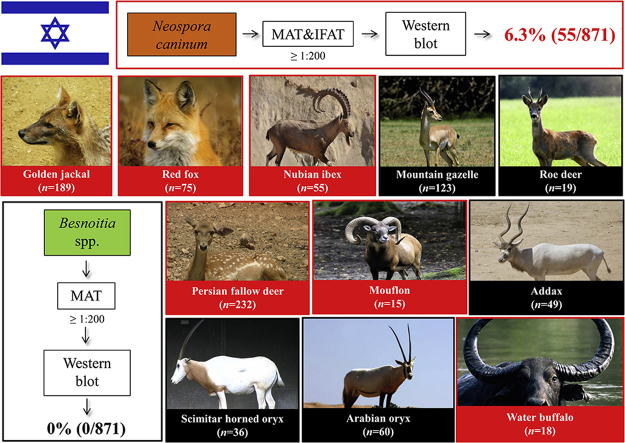

Neosporosis and besnoitiosis are protozoan reproductive and productive diseases of cattle and are caused by *Neospora caninum* and *Besnoitia besnoiti*, respectively (Dubey et al., 2017; Alvarez-García et al., 2013). Both diseases are present in Israel (Fish et al., 2007; EFSA, 2010). Specifically, more than 45% of cows in dairy farms were seropositive to *N. caninum*, and *Neospora*-associated abortions have also been reported (Fish et al., 2007). Bovine besnoitiosis was widely reported in the 1960s; however, the current epidemiological situation is unknown even after several decades of using a live tachyzoite vaccine from an Israeli bovine isolate (unpublished data).

Although domestic cattle act as intermediate host for both parasites, both infections have also been diagnosed in wild animal species. Dogs, dingoes, coyotes and grey wolves are definitive hosts for *N. caninum*, whereas the definitive host for *B. besnoiti* is still unknown despite the fact that both, domestic and wild cats, have been suggested (Alvarez-García et al., 2013; Dubey et al., 2017). Nevertheless, the putative role of a sylvatic life cycle in the epidemiology of both diseases in cattle has not been fully elucidated (Gondim, 2006; Alvarez-García et al., 2013). The location of Israel at the border of four biogeographical regions contributes to the abundance of a wide range of wild animals. However, the search for specific *N. caninum* antibodies has been restricted to wild carnivores and crows (Steinman et al., 2006; Salant et al., 2015).

The detection of specific antibodies in wildlife is a challenge due to: i) the use of non-validated serological tools and the absence of reference sera (Gutiérrez-Expósito et al., 2016; Bartova et al., 2017); ii) low sample quality that leads to the degradation of immunoglobulins; iii) lack of species-specific secondary antibodies and iv) cross-reactions with closely related parasites. Therefore, Donahoe et al. (2015) suggested the use of more than one serological technique to obtain accurate results.

In the present study, we evaluated the presence of specific antibodies against *Neospora* spp. and *Besnoitia* spp. parasites in a wide panel of wild animals. Three serological assays were used to detect *Neospora* spp. and, two were used for *Besnoitia* spp.

A total of 871 samples from two wild carnivorous species and nine ruminant species were analysed (Table 1). Between 2006 and 2013, blood samples were collected from animals during regular monitoring and from dead animals at different locations in North, Central and South Israel by the Nature and Parks Authorities of the country (Fig. 1). Halula preservation (area A) and Hai-Bar Nature Reserve (area B) are fenced areas located in the north, whereas areas C (Ein Gedi Reserve), D (Ein Hazeva) and E (Yotvata) are open areas located in central and southern Israel. Age and sex data for sampled animals were unavailable. Most of samples from wild carnivores were collected from dead animals whereas the majority of samples from wild ruminants were from live animals. The serum was obtained by centrifugation and maintained at −80 °C until tested. All sera were initially analysed by the modified agglutination test (MAT) (Packham et al., 1998; Waap et al., 2011). In addition, a complementary immunofluorescence antibody test (IFAT) was used for the detection of specific *N. caninum* antibodies (Fish et al., 2007). Subsequently, samples with MAT and/or IFAT titres ≥1:200 were posteriorly confirmed using *N. caninum* and *B. besnoiti* tachyzoite-based Western blot as a gold standard technique (Alvarez-García et al., 2002; García-Lunar et al., 2013). An animal was considered positive if the presence of specific antibodies was confirmed by Western blot analysis.

**Table 1:** Detection of specific anti-*Neospora* antibodies by Western blot in IFAT and/or MAT positive sera.

Species		Samples (n)	MAT^a^ (%, nº positive/nº tested)	IFAT^a^ (%, nº positive/nº tested)	Western blot confirmation (%, nº positive/nº sample tested)^b^		Seroprevalence (%)^c^
					MAT	IFAT	
Carnivores	Golden jackal (*Canis aureus*)	189	**15.3 (29/189)**	**2.1 (4/189)**	**20 (6/29)**	**50 (2/4)**	**3.2 (6/189)**
	Red fox (*Vulpes vulpes*)	75	**12.0 (9/75)**	**1.3 (1/75)**	**11.1 (1/9)**	**100 (1/1)**	**1.3 (1/75)**
Ruminants	Addax (*Addax nasomaculatus*)	49	**24.4 (12/49)**	0.0 (0/49)	0.0 (0/12)	–	0.0 (0/12)
	Arabian oryx (*Oryx leucoryx*)	60	**5.0 (3/60)**	0.0 (0/60)	0.0 8 (0/3)	–	0.0 (0/3)
	Persian fallow deer (*Dama mesopotamica*)	232	**23.3 (54/232)**	**5.17 (12/232)**	**24.1 (13/54)**	**100 (12/12)**	**5.6** (**13/232**)
	Mouflon *(Ovis orientalis)*	15	**40 (6/15)**	**6.7 (1/15)**	**16.7 (1/6)**	**100 (1/1)**	**6.7 (1/15)**
	Mountain gazelle (*Gazella g. gazella*)	123	**18.7 (26/123)**	**2.4 (3/123)**	0.0 (0/26)	0.0 (0/3)	0.0 (0/123)
	Nubian ibex (*Capra nubiana*)	55	**65.4 (36/55)**	**27.3 (15/55)**	**61.1 (22/36)**	**100 (15/15)**	**40.0 (22/55)**
	Roe deer (*Capreolus capreolus*)	19	0.0 (0/19)	0.0 (0/19)	–	–	0.0 (0/0)
	Scimitar horned oryx (*Oryx dammah*)	36	**2.7 (1/36)**	0.0 (0/36)	0.0 (0/1)	–	0.0 (0/1)
	Water buffalo (*Bubalus bubalis*)	18	**77.8 (14/18)**	**72.2 (13/18)**	**85.7 (12/14)**	**84.6 (11/13)**	**66.7 (12/18)**
***Total***		*871*	*21.8 (190/871)*	*5.9 (52/871)*	*18.4 (35/190)*	*85.7 (42/49)*	*6.3 (55/871)*

a Antibody titer equal or higher than 1:200.

b Only positive result by either IFAT or MAT were tested by WB.

c Number of WB-positive animals/total of sampled animals.

**Fig. 1:**
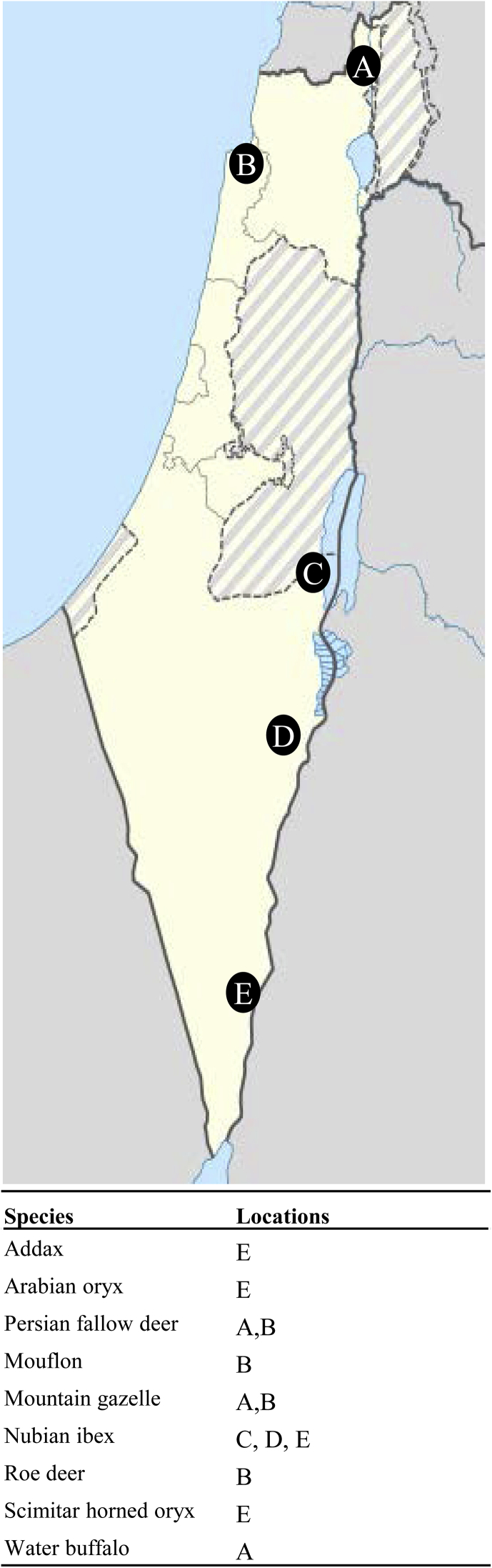
**Geographical distribution of wild ruminants sampled in Israel. The places showed only ruminants with total number of samples higher than 15**. Area A: Fenced Halula preservation. Area B: Fenced Hai-Bar Nature Reserve. Area C: Ein Gedi Reserve. Area D: Ein Hazeva. Area E: Yotvata.

Tachyzoites of the Nc1 *N. caninum* isolate (Dubey et al., 1988) and BbSp-1 isolate of *B. besnoiti* (Fernández-García et al., 2009) were grown *in vitro* as previously described (Fernández-García et al., 2009). Later, these tachyzoites were pelleted and frozen at −80 °C for Western blot tests or resuspended in PBS and formalin-fixed for IFAT and MAT.

*Neospora* spp.-based MAT was carried out as previously described (Packham et al., 1998). Sera were diluted serially two-fold from 1:100 to a final dilution of 1:12,800. Due to the lack of a reference panel of sera and to avoid overestimating positive results, a conservative cut off of 1:200 was selected. Two positive bovine sera control samples, one negative bovine sera control sample and a single non-serum control sample were included on each plate. The presence of *Neospora* spp. antibodies by IFAT was carried out according to http://www.sciencedirect.com/science/article/pii/S0304401715004021, Fish et al. (2007) by using FITC-labelled rabbit anti-dog, anti-sheep, anti-bovine, anti-deer and anti-goat secondary antibodies at a concentration of 1:60 for canids and 1:80 for mouflon, *Bovinae* species, *Cervidae* species and Nubian ibex. The highest dilution at which the whole parasites showed fluorescence was considered as the endpoint titre.

MAT for *Besnoitia* spp. was performed as previously described (Waap et al., 2011) with a few modifications: the initial serum dilutions were 1:100 and 1:200, and tachyzoites were resuspended to a final concentration of 40,000 tachyzoites/μL. A well-coded panel of 125 ruminant sera from cattle (n = 42 seropositive and n = 39 seronegative) (Gutiérrez-Expósito et al., 2017a), 35 from caribou (n = 15 seropositive and n = 20 seronegative) (Gutiérrez-Expósito et al., 2012) and 5 from sheep (n = 5 seronegative) (Gutiérrez-Expósito et al., 2017b) tested by Western blot were used to standardize an *in-house* MAT. Two samples from seropositive animals with dermal cysts and two samples that were seronegative by IFAT and Western blot from a non-endemic area were included in each plate. The dilution with the best values of sensitivity (Se) and specificity (Sp) was selected as a cut off.

*Neospora caninum* and *B. besnoiti* tachyzoites were processed and Western blots were carried out under reducing and non-reducing conditions as previously described (Alvarez-García et al., 2002; García-Lunar et al., 2013). Tachyzoites were exposed to sera from positive-IFAT and/or MAT animals using a 1:20 dilution and a second incubation step with Protein A–Peroxidase-labelled (P8651, Sigma) diluted at 1:200 was used for carnivorous species (golden jackal and red fox). Rabbit peroxidase-labelled anti-deer IgG (H + L) antibody conjugate (04-31-06 KPL, Gaithersburg, MD, USA) diluted at 1:200 was used for *Cervidae* sera (Persian fallow deer and roe deer), and protein G (Recombinant-Peroxidase Labeled, Sigma^®^), at a 1:500 dilution was used for *Bovidae* sera (addax, Arabian oryx, mouflon, mountain gazelle, Nubian ibex, scimitar horned oryx and water buffalo). For the *Neospora* spp.-based Western blot, the presence of the immunodominant 17–18 kDa antigen was the criterion for a positive result (Álvarez-García et al., 2002), whereas for the *Besnoitia* spp.-based Western blot, the criterion described by García-Lunar et al. (2013) was considered as a positive result.

*Neospora* spp. antibodies were found in golden jackal, red fox, addax, Arabian oryx, Persian fallow deer, mouflon, mountain gazelle, Nubian ibex, scimitar horned oryx and water buffalo by IFAT and/or MAT (Table 1). A cut-off of 1:200 was selected for both tests as only four out of 26 sera with an IFAT titre of 1:100 were confirmed by Western blot (data not shown) and none of the sera with a MAT titre of 1:100 (n = 157, data not shown) could be confirmed by IFAT. Specifically, 52 samples were seropositive by both serological techniques, and 141 were seropositive only by MAT. The highest antibody levels (≥1:800) were found in golden jackal, Persian fallow deer, mountain gazelle, Nubian ibex and water buffalo (Table 2). However, the presence of *Neospora* spp.-specific antibodies was confirmed by Western blot only in golden jackal (6/189; 3.2%), red fox (1/75; 1.3%), Persian fallow deer (13/232; 5.6%), mouflon (1/15; 16.7%), Nubian ibex (22/55; 40%) and water buffalo (12/18; 66.7%) (Table 1) (Fig. 2). MAT-positive sera were confirmed in 18.6% samples with a titre of 1:200, in 14.5% samples with a titre of 1:400, in 57.1% samples with a titre of 1:1600 and in 100% sera with a titre of 1:6400 by Western blot. Nevertheless, the recognition of immunodominant bands by Nubian ibex and mouflon was weak (Fig. 2). In addition, animal species with a sample size lower than 15 were also analysed together with the rest of samples but not included in this study: badger (*Meles meles*) (*n* = 7), caracal (*Caracal caracal*) (n = 2), grey wolf (*Canis lupus*) (n = 9), hyena (*Hyaena hyaena*) (n = 10), jungle cat (*Felis chaus*) (n = 1), leopard (*Pantera pardus*) (n = 2), marten (*Marten foina*) (n = 2), wild cat (*Felis silvestris*) (n = 2), Acacia gazelle (*Gazella gazella acaciae*) (n = 1), Cretan ibex (*Capra aegagrus creticus*) (n = 9), Dorca gazelle (*Gazella dorcas*) (n = 11), eland (*Taurotragus oryx*) (n = 1) and red deer (*Cervus elaphus*) (n = 3). *Neospora* specific antibodies were found in the only sampled eland, one out of three red deers and one out of ten hyenas by Western blot (data not shown). Thus, this work indicates the need of employing at least two complementary diagnostic tests to obtain more accurate results.

**Table 2:** Anti-Neospora antibody titers in IFAT positive sera.

	IFAT titers						
	1/200	1/400	1/800	1/1600	1/3200	1/6400	1/12800
Golden jackal	0/4	1/4	1/4	1/4	–	–	–
Red fox	–	–	–	–	1/1	–	–
Persian fallow deer	–	–	–	–	1/12	3/12	8/12
Mouflon	1/1	–	–	–	–	–	–
Mountain gazelle	3/3	–	–	–	–	–	–
Nubian ibex	10/18	3/18	4/18	1/18	–	–	–
Water buffalo	1/13	5/13	2/13	4/13	1/13	–	–

**Fig. 2:**
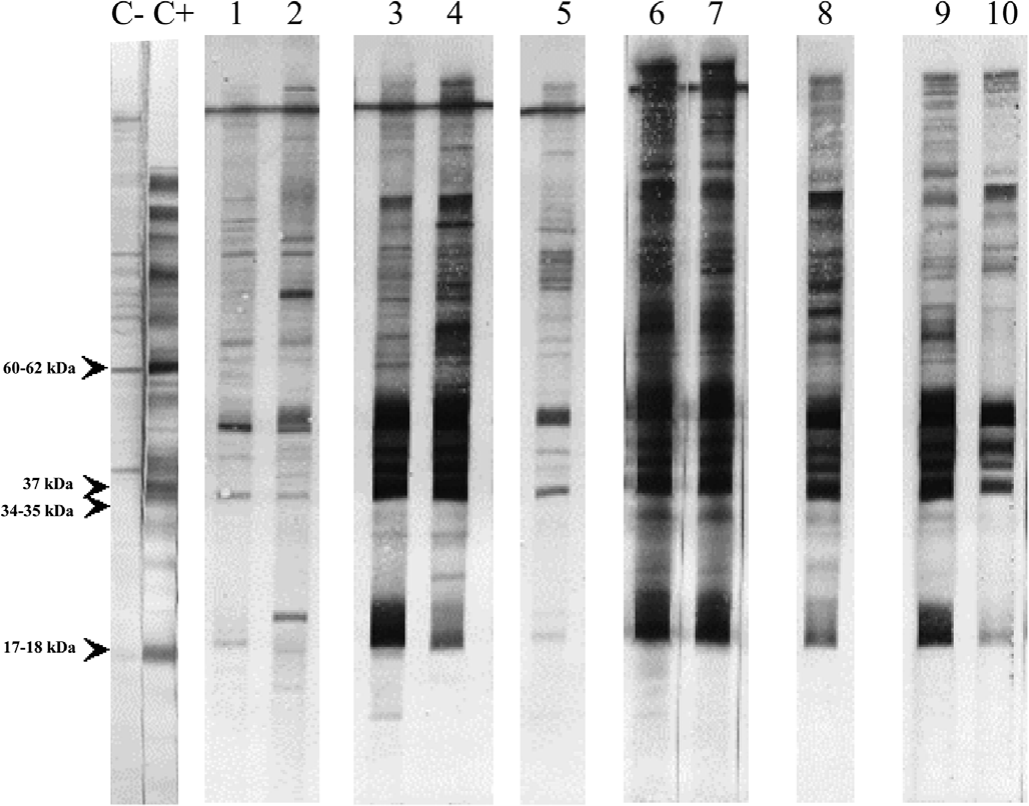
**Recognition of *Neospora* spp. tachyzoite antigens by Western blot in sera from wildlife**. C-: Negative control serum from a cow. C+: Positive control serum from a *N. caninum* infected cow. Lines 1–2: Nubian ibex. Lines 3–4: Water buffalo. Line 5: Mouflon. Lines 6–7: Persian fallow deer. Line 8: Red fox. Lines 9–10: Golden jackal. Arrow indicates recognition of immunodominant antigens (IDAs) described by Alvarez-García et al. (2002).

For *Besnoitia* spp. sero-survey Se and Sp values were calculated for MAT titres of 1:100, 1:200 and 1:400 on the basis of the results of the well-coded panel sera tested by Western blot. The cut off of 1:100 yielded 100% Se and 75% Sp in contrast to 93.0% Se and 88.2% Sp corresponding to a 1:200 cut off. Additionally, the cut off of 1:400 yielded 75.4% Se and 97% Sp. Thus, the cut off of 1:200 was selected. MAT-seropositive animals were found only in water buffalo (*n* = 4) with a titre of 1:200 but were negative by Western blot. Carnivorous species were all seronegative by MAT.

We ran the most comprehensive serological survey to date for *Neospora* specific antibodies in Israel. Additionally, this is the first sero-survey of *Besnoitia* spp. infection in wildlife in this country. Antibodies against *Neospora* were detected in a low number of animals of four species in contrast to a relative high seroprevalence observed in both Nubian ibex (40%) and water buffalo (66.7%). The differences observed in *N. caninum* seroprevalence among the wild ruminant species are unlikely to be due to the area where they graze. In fact, high and low seroprevalence levels were reported in areas A and B (Fig. 1), whereas positive Nubian ibex and negative addax, Arabian oryx and Scimitar horned oryx were sampled in areas C, D and E (Fig. 1).

Antibodies against *Besnoitia* spp. were not found despite the fact that the disease has been widely reported in domestic cattle in Israel (Bigalke, 1981) and few bovine cases are sporadically diagnosed (unpublished data). Concerning the methods used in this study, several authors performed different serosurveys in wildlife using one or more serological techniques, and the seroprevalence notably varied depending on experimental design, animal species, sample size, geographical area and laboratory tests employed (reviewed by Donahoe et al., 2015 and Dubey et al., 2017). Thus, a conservative diagnostic approach using two or three tests was followed herein. Moreover, Western blot is the serological technique recommended for the confirmation of uncertain results in cattle (García-Lunar et al., 2013; Guido et al., 2016). Accordingly, two complementary tests were previously employed in a few *Besnoitia* serosurveys carried out in wild carnivores and ruminants in Europe (Millan et al., 2012; Gutiérrez-Expósito et al., 2016).

For the *Neospora* serosurvey, MAT appeared to have a high sensitivity but low specificity as only 18.4% of the MAT positive samples with titres of 1:200 were confirmed by Western blot. This finding showed that low MAT-positive titres should be interpreted cautiously in the absence of a second/confirmatory test. The low specificity, observed in MAT-positive results, could be the result of cross-reactions with closely related Sarcocystidae parasites such as the *Toxoplasma gondii* or *Sarcocystis* spp. (Gondim et al., 2017). IFAT showed better diagnostic accuracy since 42 out of 49 seropositive results were successfully confirmed by Western blot. Thus, our results revealed a discrepancy between MAT and IFAT. The lack of a test validation with appropriate reference sera for each animal species is an important MAT disadvantage (Gondim et al., 2017).

The seroprevalence of *N. caninum* for water buffalo was 66.7%. Reichel et al. (2015) suggested that water buffalo might be a more common intermediate host than cattle. Water buffalo is a relevant livestock species in several Asian, African, Oceanian and South-American countries such as India, Iran, Pakistan, Kenya, Egypt, Argentina and Brazil, where its seroprevalence varies from 42.2% to 88% (Konrad et al., 2013; Neverauskas et al., 2015). Further studies should estimate the impact of *Neospora* spp. infection in water buffalo. Moreover, we have detected, for the first time, anti-*Neospora* spp. antibodies in Nubian ibex, an endangered goat located in the mountainous desert areas of northeast Africa and parts of Arabia. Although the epidemiology and economic importance of caprine neosporosis in domestic goats remains poorly investigated, the seroprevalence varies from 1% to 23% (Dubey and Schares, 2011), and recent studies considered *N. caninum* as an important abortifacient in small ruminants (Moreno et al., 2012). Nevertheless, the studies of *N. caninum* infection in free-ranging wild goats are limited to Alpine and Spanish ibex (*Capra ibex* and *Capra pyrenaica hispanica*, respectively) in which seroprevalence was 1.4% and 5.1%, respectively (Almería, 2013). Additionally, antibodies in Persian fallow deer and mouflon were detected in a scarce number of samples, which was similar to the very low seroprevalence previously reported in Europe for these species (1% and 3%, respectively) (Bartova et al., 2007). The lack of antibodies in roe deer contrasts with the seroprevalence described in European studies which varies from 2.7% to 14% (Almería, 2013). Ideally, the number of roe deer sera should be increased in further studies. However, it may not be an easy task since roe deer is not a widespread species in Israel. Furthermore, the results obtained from wild canids species support the findings of Steinman et al. (2006) in Israel, who detected a low exposure to *N. caninum* (4 out of 147) by IFAT in golden jackals, foxes and wolves. Thus, the contact of sampled carnivores with infected tissue from ruminants or small mammals was infrequent. It has been postulated that the exposure of carnivores to the parasite varies in different habitats depending on the intermediate host species consumed (Stuart et al., 2013). A greater sample size would be needed to confirm the absence or low exposure in the specific species studied and in those particular species whose sample size was low. Overall, serology is a good tool to detect *N. caninum* antibodies in carnivores that faces several limitations. First of all, the quality of the sera collected from dead animals can affect the antibody detection. For this reason complementary serological tests were carried out as suggested by Donahoe et al. (2015). Secondly, seropositivity only indicates exposure to the parasite. The detection of oocysts is needed in order to confirm a species as definitive host of *N. caninum*, and, it has been proven that seronegative dogs can shed *N. caninum*-oocysts (Gondim, 2006; Cavalcante et al., 2011).

*Besnoitia* spp. infection was also studied in wildlife in Spain (Millán et al., 2012; Gutiérrez-Expósito et al., 2016). Although red deer and roe deer can be seropositive, their role in the epidemiology of bovine besnoitiosis is of limited importance (Gutiérrez-Expósito et al., 2016). Similarly, the lack of antibodies against *Besnoitia* spp. for 16 carnivore species from Spain suggested their unlikely implication in the parasite transmission at least as intermediate hosts (Millán et al., 2012).

In summary, the high seroprevalence of specific *Neospora* antibodies found in water buffalos and Nubian ibex are indicative of a high exposure of these wild herbivores to the parasites, which was similar to the high seroprevalence observed in cattle in Israel. However, the source of infection should be further examined as low seroprevalence was observed in all wild carnivores examined. Concerning besnoitiosis, it appears that wild animals might not present a significant threat for livestock infections in Israel. However, these studies help to identify putative hosts or reservoirs in wildlife that are present in different habitats, and sampling wild carnivore species would help to identify the putative definitive host. Nevertheless, further studies with a larger sample size focused on the species that have proven to be seropositive might be considered. Finally, since the Nubian ibex is an endangered species, the study of infectious and parasitic diseases would improve the understanding of the epidemiology and the impact of these diseases on this ruminant species.

## Conflicts of interest

The authors have no conflicts of interest.

## Appendix A Supplementary data

Data Profile Data Profile
